# Response Surface Methodology-Guided Development of Hot-Melt-Extruded Cannabidiol Solid Self-Emulsifying Drug Delivery Systems for Enhanced Drug Release and Stability

**DOI:** 10.3390/pharmaceutics18101302

**Published:** 2026-10-14

**Authors:** Kavish Sanil, Janhavi Deshmukh, Eman A. Ashour

**Affiliations:** Department of Pharmaceutics and Drug Delivery, School of Pharmacy, University of Mississippi, University, MS 38677, USA; ksanil@go.olemiss.edu (K.S.); jdeshmuk@go.olemiss.edu (J.D.)

**Keywords:** cannabidiol (CBD), self-emulsifying drug delivery system (SEDDS), hot-melt extrusion, response surface methodology, drug release, stability

## Abstract

**Background/Objectives**: Cannabidiol (CBD) is a non-psychoactive phytocannabinoid, with numerous therapeutic applications. It can be used to treat pain, inflammation, anxiety, and schizophrenia and it is also used to treat severe forms of infant epilepsy. CBD is a highly lipophilic compound with low oral bioavailability due to poor aqueous solubility and extensive first-pass metabolism. Self-emulsifying drug delivery systems (SEDDSs) offer a promising approach to enhancing the solubility and stability of lipophilic compounds like CBD. This study aimed to design and optimize CBD-loaded solid SEDDSs (S-SEDDSs) using Response Surface Methodology (RSM) to identify the optimal formulation parameters for improved drug release and stability. **Methods**: Excipients were selected through qualitative screening studies, considering their functional properties in SEDDS development. A Central Composite Design (CCD) was employed for response surface optimization to evaluate the effect of independent variables on formulation performance, including oil concentration and the surfactant–co-surfactant ratio. Formulations were processed using hot-melt extrusion (HME). The lead formulation was tested for drug content, in vitro drug release and stability studies. **Results**: The solid SEDDS formulation showed a drug content of 98.15 ± 1.94% and achieved 76.6 ± 0.6% drug release within three hours during in vitro dissolution testing, compared to 28 ± 0.9% for pure CBD. During stability testing, the lead formulation showed minimal change at both 25 °C and 40 °C. **Conclusions**: Overall, the study demonstrated that RSM-guided formulation development combined with HME processing can produce CBD S-SEDDSs and improve in vitro drug release compared to pure CBD.

## 1. Introduction

Oral delivery is the most commonly and widely used route for drug delivery due to its ease of administration, high patient compliance, minimal sterility requirements, cost-effectiveness, and overall versatility [1]. Approximately 80% of pharmaceutical products available commercially are delivered through the oral route [2]. More than half of the drugs currently under development have been estimated to have low aqueous solubility, leading to poor drug release and efficacy [3]. In recent decades, numerous formulation techniques have emerged to enhance the oral bioavailability of poorly water-soluble drugs by improving their apparent solubility in gastrointestinal fluids [4].

Cannabidiol (CBD) is a non-psychoactive cannabinoid derived from the plant *Cannabis sativa* L. [5], and has emerged as a promising therapeutic agent with several potential therapeutic applications [6,7,8,9,10]. However, oral administration of CBD faces challenges because of its poor aqueous solubility, high lipophilicity and extensive first-pass metabolism, which contribute to CBD’s low and variable oral bioavailability [11].

To tackle these challenges, a variety of approaches can be applied, including lipid-based drug delivery systems (LBDDSs), polymeric nanoparticulate systems, cyclodextrin complexation, and co-crystal technology [12,13,14,15].

Among LBDDS, self-emulsifying drug delivery systems (SEDDSs) have received a lot of attention lately because of their ability to improve the dispersion and solubility of lipophilic drugs [16,17]. SEDDSs are isotropic mixtures of an oil or lipid, a surfactant, and a co-surfactant or co-solvent [18,19] that produce a fine oil-in-water (o/w) emulsion following slight agitation generated by peristaltic movement, followed by dilution in the gastrointestinal (GI) fluid [20,21]. Commercially marketed SEDDSs are liquids contained in either a hard gelatin capsule (Gengraf^®^, Lipirex^®^) or a soft gelatin capsule (Sandimmune^®^, Neoral^®^) [22]. There are a few disadvantages to liquid SEDDSs, which limit the number of commercially viable products. They require encapsulation in soft gelatin capsules, which are expensive, have issues with stability and can lead to precipitation of the drug [23,24]. One strategy to overcome these challenges involves formulating solid dosage forms of SEDDSs. This approach not only enhances the formulation’s stability but also improves drug absorption and release [25]. Established methods for solidifying liquid SEDDSs include spray drying, freeze drying, and adsorption onto solid carriers [26]. Hot-melt extrusion (HME) is mainly used in the production of amorphous solid dispersions, as it is a solvent-free technique and is easily scalable to continuous manufacturing [27,28]. The technique allows for the melting and mixing of liquid or powdered raw material to produce an intermediate or final solid dosage form [29]. HME is highly adaptable; the screw configuration can be modified to fit the specific needs of the processed formulation, and one or more feeding zones can be used to introduce the physical mixture into the system via powder or liquid feeders [30]. HME has been investigated as an effective means of producing solid SEDDSs [31,32]. CBD’s poor aqueous solubility and extensive first-pass effect make it an ideal candidate for formulation development using S-SEDDSs.

To build a successful S-SEDDS, the ratios between the oil, surfactant, co-surfactant, and solid carrier must be properly selected to ensure optimum drug loading [33]. Using Response Surface Methodology (RSM), a statistical and mathematical tool, can provide a systematic and efficient approach to optimization by investigating the links between different formulation factors and their effects on key quality parameters [34]. Within RSM, the Central Composite Design (CCD) is particularly valuable because it enables efficient exploration of factor interactions and curvature, supporting accurate prediction of optimal formulation conditions. A Central Composite Design (CCD) is made up of three primary components: factorial (or fractional) points, axial or “star” points, and center points. The axial points are set at a specific distance (α) from the design center, expanding the experimental space beyond the basic factorial design. This structure allows for accurate calculation of curvature and quadratic effects, resulting in a greater understanding of the response surface [35].

In our previous study [31], the feasibility of using HME to prepare CBD-loaded S-SEDDSs was demonstrated using formulations containing sesame oil, Poloxamer 188, Gelucire^®^ 59/14, and polymeric carriers (PEO N80 and Soluplus^®^), with the lead formulations showing droplet sizes of 185–366 nm, PDI values of 0.10–0.39, and emulsification times of approximately 9 min. Although these formulations substantially improved CBD’s dissolution compared to the pure drug, formulation development was based primarily on preliminary screening, and the effects of individual formulation variables were not evaluated. The formulations changed significantly during the stability study, undergoing both physical and chemical changes. The present study builds on this work using a different S-SEDDS composition containing sesame oil, Gelucire^®^ 50/13, PEG 300, and Neusilin^®^ US2 and, importantly, applies RSM using a CCD to systematically investigate the effects of oil concentration and surfactant-to-co-surfactant ratio on particle size, emulsification time, and PDI. This approach enabled optimization and validation of the selected formulation, providing a better understanding of the relationship between formulation composition and the performance of HME-processed CBD S-SEDDSs.

## 2. Materials and Methods

### 2.1. Materials

The cannabidiol (CBD) used in this study was kindly gifted by ElSohly Laboratories, Inc. (Oxford, MS, USA). Sesame oil and PEG 300 were obtained from Fisher Scientific (St. Louis, MO, USA). Gelucire^®^ 50/13 was received as a gift from Gattefossé (Paramus, NJ, USA), and Neusilin^®^ US2 was obtained from Fuji Chemical Industries (Burlington, NJ, USA). All solvents used for analysis were HPLC grade and purchased from Fisher Scientific (St. Louis, MO, USA).

### 2.2. Methods

#### 2.2.1. Excipient Screening Studies

A preliminary excipient screening was performed to select the oil, surfactant and co-surfactant for further formulation development. A fixed amount of CBD (100 mg) was mixed with 1000 mg of each excipient to be screened. Each drug–excipient mixture was heated and stirred at 80 ± 0.5 °C and 1000 rpm for 15 min, then allowed to cool to room temperature. Samples were visually inspected for phase separation or precipitation of the drug. Excipients that showed no evidence of precipitation and miscibility with CBD were selected for further formulation development, together with their functional properties and previously reported use in the development of SEDDSs. The screening was not intended to determine the equilibrium or saturation solubility of CBD in the individual excipients.

#### 2.2.2. Experimental Design

Using the data obtained from the preliminary formulation screening and supporting literature, two key formulation variables that affected SEDDS performance were chosen for optimization. The variables included oil concentration (X1) and surfactant–co-surfactant ratio (X2); the ratio of co-surfactant was set to 1, while the ratio of surfactant varied according to the experimental design. The oil concentration and surfactant–co-surfactant ratio were selected based on our previous formulation study [31] and the supporting literature [32,36]. Oil concentrations between 10 and 20% *w*/*w* have been shown to have faster self-emulsification and better thermodynamic stability in SEDDSs in comparison to formulations having 30–40% *w*/*w* oil concentration [36].

Rather than constructing a conventional pseudo-ternary phase diagram, the present study employed a CCD-based response surface approach to evaluate the composition-dependent self-emulsification behavior within the predefined design space relevant to HME processing. The design enabled quantitative assessment of the effects of oil concentration and surfactant-to-co-surfactant ratio on particle size, emulsification time, and PDI. Accordingly, the purpose of the experimental design was to identify an optimized composition based on measured self-emulsification performance rather than defining the complete phase behavior of the corresponding liquid oil/surfactant/co-surfactant system.

As a result, 10–20% *w*/*w* sesame oil was chosen as the factorial range in the current study. CCD was used to study the influence of these factors on particle size (Y1), emulsification time (Y2), and polydispersity index (Y3). The selected factors—oil concentration (% *w*/*w*, X1) and surfactant–co-surfactant ratio (X2)—were examined at five levels: the center point (X1: 15% *w*/*w*, X2: 3), level +1 (X1: 20% *w*/*w*; X2: 4.0), level −1 (X1: 10% *w*/*w*; X2: 2.0), level +α (X1: 22.0711% *w*/*w*; X2: 4.41421), and level −α (X1: 7.92893% *w*/*w*; X2: 1.58579). The axial distance (α = 1.414) was determined using the equation ±√k for k = 2 factors. The CCD was generated using Design-Expert^®^ software (Stat-Ease^®^, Minneapolis, MN, USA, version 13), resulting in 11 experimental runs that included factorial points, axial points, and replicated center points to estimate pure error. The runs were randomized to reduce systematic bias and ensure robustness of the model. A summary of the experimental design and factor settings is provided in Table 1. Other formulation and processing variables, including drug loading, carrier concentration, extrusion temperature, and screw speed, were kept constant; therefore, the resulting models and optimization are limited to the two-factor design space evaluated in this study.

#### 2.2.3. Preparation of S-SEDDSs

Accurately weighed quantities of CBD, Sesame oil, Gelucire^®^ 50/13, PEG 300, and Neusilin^®^ US2 were blended using a mortar and pestle to obtain 20 g of physical mixture (PM) for each design run. The composition of the different formulations used to make the PM’s according to the runs is provided in Table 2. The formulation component concentrations were reported as a percentage of the total formulation weight (% *w*/*w*). CBD and Neusilin^®^ US2 concentrations were kept constant at 10% and 40% *w*/*w*, respectively, throughout the design runs, accounting for 50% of the formulation. The remaining 50% *w*/*w* was made up of sesame oil, Gelucire^®^ 50/13, and PEG 300. These PMs were subsequently processed using an 11 mm hot-melt extruder (Process11, Thermo Fisher Scientific, Waltham, MA, USA). Extrusion was performed at a barrel temperature of 90 °C (zones 3–8) with a standard screw configuration containing three mixing zones and four conveying zones, used at a constant screw speed of 50 rpm for all formulations. The feeding zone was maintained at 25 °C. The physical mixture was manually fed into the extruder, and torque was monitored throughout processing and maintained at 15–20% of the machine’s capacity. The extrudates were collected, cooled to room temperature, milled, and then used for subsequent evaluations.

#### 2.2.4. Emulsification Time (ET)

For each formulation generated from the DOE, 100 mg of the S-SEDDS was dispersed in 20 mL of deionized water in a glass beaker maintained at 37.5 °C with gentle stirring at 100 rpm to evaluate its self-emulsification behavior. The ET was recorded as the time required for complete dispersion of the formulation and formation of a homogeneous emulsion. All evaluations were conducted in triplicate to ensure reproducibility across the design runs.

#### 2.2.5. Particle Size (PS), Zeta Potential (ZP) and Polydispersity Index Measurement (PDI)

Particle size (PS), polydispersity index (PDI), and zeta potential (ZP) of the formulations were measured using a Zetasizer Nano ZS (Zen3600, Malvern Panalytical Inc., Westborough, MA, USA) at 25 °C. Prior to DLS analysis, the reconstituted dispersions were filtered through Whatman filter paper to remove residual insoluble Neusilin^®^ US2, which served as the solid carrier and was not considered part of the self-emulsified lipid droplets. The resulting filtrate was diluted 100-fold with Milli-Q water before PS and PDIs. The same sample-preparation procedure was applied consistently to all formulations to permit comparison across the experimental design. It should be noted that filtration may remove some larger lipid droplets or aggregates in addition to the insoluble carrier and that dilution may alter the colloidal equilibrium of the dispersed system. Consequently, the reported PS and PDI values represent the characteristics of the filtered and diluted self-emulsified fraction under the specified analytical conditions rather than the complete particle-size distribution of the original undiluted dispersion.

#### 2.2.6. Differential Scanning Calorimetry (DSC)

Differential scanning calorimetry (DSC) was conducted using a Discovery DSC 25 (TA Instruments, Newcastle, DE, USA) equipped with an RCS90 refrigeration system. Initial measurements were performed to determine the melting points of pure CBD, the Gelucire^®^, and Neusilin^®^ US2. Subsequently, milled extrudates and physical mixtures were analyzed. Approximately 5–10 mg of each sample was placed in hermetically sealed aluminum pans and equilibrated for 1 min before heating from 25 °C to 250 °C at a ramp rate of 10 °C/min under an ultrapure nitrogen purge (50 mL/min). Heat flow versus temperature curves were recorded and processed using Trios software, TRIOS V6.0 (TA Instruments, New Castle, DE, USA).

#### 2.2.7. Powder X-Ray Diffraction (PXRD)

PXRD analysis was performed to evaluate the crystallinity of the prepared SEDDS formulation. Diffractograms of pure CBD, Gelucire^®^ 50/13, Neusilin^®^ US2, the physical mixture, and the optimized formulation (F7) were recorded at room temperature using a Bruker D6 PHASER X-ray diffractometer (Bruker, Madison, WI, USA) equipped with a Cu Kα radiation source (λ = 1.5406 Å) set to 40 kV and 15 mA. The data was collected over a 2θ range of 5–70° using continuous scanning with a step size of 0.02°.

#### 2.2.8. Fourier Transform Infrared (FTIR) Analysis

FTIR testing was used to characterize pure CBD, individual excipients, physical mixtures, and the final formulation was assessed using a Cary 630 FTIR Spectrometer equipped with a Cary FTIR ATR accessory (Agilent Technologies, Santa Clara, CA, USA) featuring a single-bounce, diamond-coated ZnSe crystal. Approximately 10 mg of sample was placed on the ATR crystal and compressed with a high-pressure clamp to ensure uniform contact. Spectra were collected in transmittance mode over the range of 600–4000 cm^−1^, using 32 scans at a resolution of 8 cm^−1^.

#### 2.2.9. Drug Content

Drug content analysis was performed for the lead formulation to confirm content uniformity and accurate drug loading. Accurately weighed samples equivalent to 25 mg ± 5 mg of CBD (n = 3) were collected from random portions of the batch and transferred into 20 mL scintillation vials. Each sample was mixed with 20 mL of acetonitrile and sonicated for 5 min to ensure complete dissolution. The resulting solutions were subsequently diluted ten times and centrifuged to remove any residual insoluble material prior to CBD quantification by high-performance liquid chromatography (HPLC). The quantification was done using a Waters HPLC-UV system (Waters Corporation, Milford, MA, USA) and analyzed using Empower 3 software. A Phenomenex (Torrance, CA, USA) Luna column (5 µm C18 (2) 100 Å, LC, 150 × 4.6 mm) was used. The mobile phase consisted of water, acetonitrile and formic acid in the ratio of 77:23:0.1(%*v*/*v*), respectively, at a flow rate of 1 mL/min. The wavelength of detection was 220 nm and calibration curves were made with concentrations ranging from 1 to 100 µg/mL [37]. The HPLC method was used for quantitative determination of CBD content. It was not specifically validated as a stability-indicating method through forced-degradation studies; therefore, the analysis was used to assess the amount of CBD rather than to establish the absence of degradation products.

#### 2.2.10. In Vitro Dissolution Testing

The in vitro release profiles of the optimized S-SEDDS formulation and pure CBD were evaluated using a USP Dissolution Apparatus II (Erweka DT 128 Light, Erwrka, Edison, NJ, USA). As no compendial USP dissolution method specific to CBD was identified, the dissolution conditions were selected based on the physicochemical properties of CBD and previously reported dissolution studies of CBD formulations [38,39]. For testing, 300 mg of the S-SEDDS formulation F7, equivalent to 30 mg of CBD, was manually filled into size 1 hard gelatin capsules without the addition of any filler or diluent. Size 1 capsules were selected because they provided sufficient capacity to accommodate the 300 mg of F7. For comparison, 30 mg pure CBD was also filled into capsules of the same size. Dissolution studies were performed in 900 mL of phosphate buffer (pH 7.4) containing 0.1% Tween 80, maintained at 37 °C and stirred at 100 rpm. Due to the poor aqueous solubility and lipophilic nature of CBD, Tween 80 was added to the dissolution media to improve the solubility and wettability of CBD. The addition of surfactants in dissolution media for poorly water-soluble drugs like CBD during in vitro analysis has been reported in the literature [39,40]. Sink conditions were not specifically evaluated for CBD in the selected dissolution medium. Aliquots were collected at predetermined intervals (5, 15, 30, 45, 60, 90, 120, and 180 min), with each withdrawn volume replaced by an equal amount of fresh medium. Samples were centrifuged at 13,000 rpm for 10 min, and the resulting supernatant was analyzed for CBD content using HPLC.

#### 2.2.11. Short-Term Stability Testing

CBD stability within the optimized S-SEDDS formulation was evaluated at two different storage conditions, 25 °C, 40 °C storage conditions over a 90-day period. Samples were collected at 30, 60, and 90 days and analyzed for CBD content. PS, PDI, ZP, and dissolution profiles were also evaluated to make sure whether the formulation retained its physicochemical integrity and performance throughout storage period.

#### 2.2.12. Statistical Analysis

This study employed Design-Expert^®^ software (StatEase^®^ Inc., Minneapolis, MN, USA, version 13.0) for formulation optimization and evaluation. The difference was considered significant when the *p*-value was <0.05.

## 3. Result and Discussion

### 3.1. Screening Studies

Preliminary excipient screening was carried out to find oils, surfactants, and co-surfactants for CBD formulation development. As summarized in Table 3, no visible phase separation or precipitation was observed in mixtures containing Tween 20, sesame oil, castor oil, Cremophor^®^ EL, Gelucire^®^ 50/13, and PEG 300. On the other hand, several of the other excipients—Propylene glycol, Poloxamer 188, Kolliphor^®^ RH 40, Comparitol ATO 888, Gelucire^®^ 44/14, Gelucire^®^ 48/16, Gelucire^®^ 59/14, Kolliphor^®^ HS 15, and Cremophor^®^ RH 40—showed apparent precipitation, suspended material, or phase separation. Since several of the tested excipients were opaque or semisolid, these findings were only used as a preliminary qualitative screening step, not as a measure of CBD solubility. Among the excipients that showed no visible precipitation or phase separation, sesame oil, Gelucire^®^ 50/13 and PEG 300 were selected for further formulation development because of their physicochemical properties and suitability for SEDDS and HME processing. Sesame oil was chosen as the oil phase based on the results of the screening study, its functional properties as a lipid vehicle and previously reported use in CBD formulations. It is used in Epidiolex^®^, a commercially available CBD oral solution which contains 100 mg/mL of the drug dissolved in sesame oil along with dehydrated alcohol, sucralose, and strawberry flavor [41]. Sesame oil was also selected because it is composed of long-chain triglycerides (LCTs), which are known to improve the solubility of highly lipophilic drugs [18]. Gelucire^®^ 50/13 was selected as the surfactant because it is amphiphilic in nature and has a high hydrophilic–lipophilic balance (HLB ~13), which is helpful in promoting self-emulsification and formation of stable emulsions [42]. It has a low melting point, which makes it suitable for HME, facilitating the processing of formulations at low temperatures [43,44]. PEG 300 was selected as the co-surfactant because of its hydrophilic nature and its ability to aid emulsion formation and dispersion in self-emulsifying systems [45].

### 3.2. Preparation of S-SEDDSs

The physical mixtures (PMs) for the formulations were prepared according to the runs provided by the design of experiments. Based on our previous study [31], which demonstrated that CBD could be processed using HME at 90 °C, all the formulations were processed at that temperature. The extrudates obtained were then cooled to room temperature before being used for further studies for the CCD. After the optimization and validation were completed, another batch of the optimized S-SEDDS was made and was used for the characterization and stability studies.

### 3.3. Statistical Analysis and Effect of Formulation Variables on S-SEDDS Responses

Data from the RSM was analyzed using Design-Expert^®^ software (Version 13) to evaluate the effects of oil concentration (X_1_) and surfactant-to-co-surfactant ratio (X_2_) on PS, PDI, and ET. ANOVA was applied to identify significant factors, interactions, and quadratic terms, and model adequacy was evaluated using R^2^ statistics, lack-of-fit tests, and diagnostic plots. The randomized experimental runs and their corresponding PS, ET and PDI responses are summarized in Table 4.

The effect of the independent factors on the PS, ET and PDI was examined using RSM-generated models. The models were statistically significant and helped in explaining the relationships between the independent factors and the measured responses.

**Figure 1:**
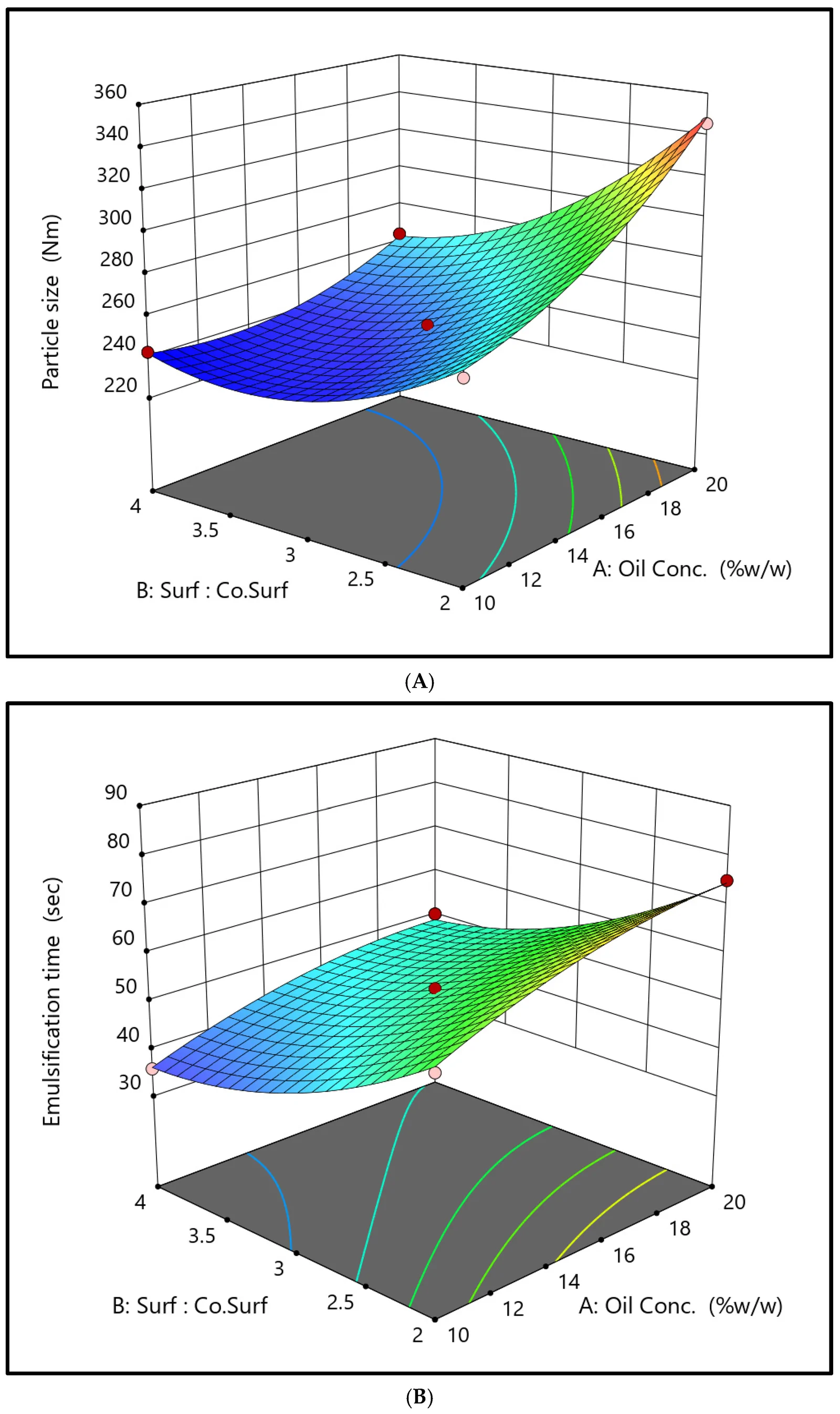

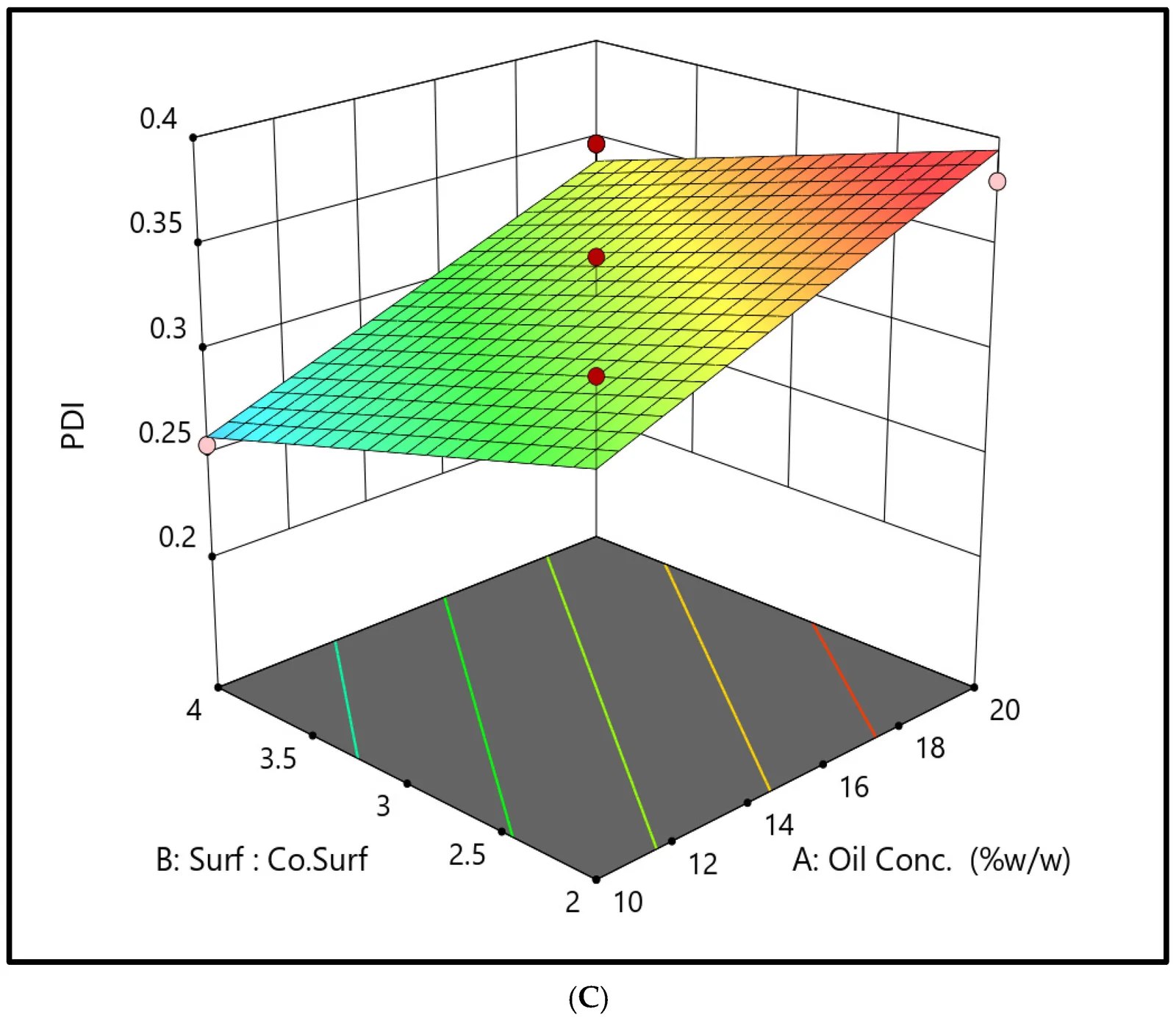
Response surface 3D plots showing the effect of oil and surfactant–co-surfactant ratio on (**A**) particle size; (**B**) emulsification time and (**C**) polydispersity index.

**Particle size**: The quadratic model for particle size was highly significant (F = 314.74, *p* < 0.0001), indicating good predictive capability. Both oil concentration (A) and surfactant–co-surfactant ratio (B) significantly influenced particle size (*p* < 0.0001). Increasing oil concentration led to a significant increase in particle size (+24.85), whereas increasing the surfactant–co-surfactant ratio reduced the particle size (–28.44) (Figure 1A). The interaction term and quadratic terms were also significant, confirming curvature effects in the response surface. The model showed a strong fit, with R^2^ = 0.9968, adjusted R^2^ = 0.9937, and predicted R^2^ = 0.9780. The adequate precision was 48.837, and the lack of fit was not statistically significant (*p* = 0.0534), supporting the adequacy of the model within the investigated design space (Table 5). The increase in PS at higher oil concentrations can be related to the greater amount of oil that has to be dispersed and stabilized in the emulsion. As the oil content increases, the surfactant may provide inadequate interfacial coating, which can cause the formation of bigger droplets and increase the chance of coalescence [46]. In contrast, increasing the surfactant ratio decreases the interfacial tension and provides better coating at the oil–water interface, allowing the formation of smaller and stable droplets [47]. This behavior was observed in the experimental formulations, with higher oil content formulations yielding bigger droplets in comparison to the formulations having lower oil content (Table 4).

**Emulsification time:** The model showed an R^2^ of 0.9963, adjusted R^2^ of 0.9926, and predicted R^2^ of 0.9472, with a CV of 2.18% and adequate precision of 50.396. The lack of fit was not statistically significant (*p* = 0.0517), supporting the adequacy of the model within the investigated design space (Table 6). ET showed a similar trend: increasing the Gelucire^®^ 50/13-to-PEG 300 ratio considerably reduced the ET, while increasing the oil concentration delayed ET (Figure 1B). The formation of emulsion is slower with higher oil concentration since a large volume of the lipid phase must be dispersed in the aqueous medium; slower dispersion might also be caused by greater viscosity linked to a large oil percentage [48], whereas increasing the proportion of surfactant can help break the oil–water interface and encourage the development of scattered droplets efficiently. As a result, the impact on particle size and emulsification time was consistent; faster emulsification and smaller droplets were often achieved under conditions that favored more efficient interfacial stability.

**Polydispersity index:** For PDI, a linear model was adequate and statistically significant (F = 16.82, *p* = 0.0014). The model showed an R^2^ of 0.8079, adjusted R^2^ of 0.7598, and predicted R^2^ of 0.5835. Although its predictive performance was lower than the particle size and emulsification time models, the adequate precision was 10.9529, and the lack of fit was not statistically significant (*p* > 0.05) (Table 7). The influence of formulation composition on PDI was less prominent than that observed for PS and ET. PDI increased modestly with increasing oil content but decreased marginally with increasing the surfactant-to-co-surfactant ratio (Figure 1C). This indicates that formulations with a higher surfactant content produced more uniform droplets, most likely due to increased interfacial stability, which inhibits droplet development and coalescence [35]. In RSM, the predictive performance of a model depends on its ability to differentiate between the measured responses and experimental variability [49]. The PDI values varied within a narrow range over the design runs (Table 4); this low variability most likely contributed to the PDI model’s lower predictive performance when compared to the particle size and emulsification timing models. As a result, the formulation factors’ influence on PDI is less significant in comparison to their more noticeable effects on particle size and emulsification time.

### 3.4. Optimization and Validation

The PS, ET and PDI are important characteristics of S-SEDDSs that define the properties of the emulsion formed when diluted with aqueous media. Emulsification time reflects how quickly the formulation forms an emulsion, while a low PDI indicates a uniform droplet distribution. Upon dilution with gentle stirring, an effective S-SEDDS formulation should disperse rapidly and uniformly.

After evaluating the results and the regression models, numerical optimization was used to determine the ideal concentration of the formulation factors. The goal was to reduce PS, ET, and PDI, while maintaining the formulation variables within the design space listed in Table 8. The optimized conditions corresponded to formulation F7, which contained 10% *w*/*w* sesame oil and a 4:1 Gelucire^®^ 50/13-to-PEG 300 ratio. These formulation characteristics were consistent with the results found in the response surface analysis, which showed that lower oil concentrations and a larger relative quantity of surfactant led to smaller particle size and faster emulsification. A new batch of F7 was then prepared using HME to validate the model, and experimentally measured responses were compared to predicted values (Table 9). The prediction errors were 0.82%, 3.27%, and 4.0% for PS, ET, and PDI, respectively, and all the values fell within the 95% confidence intervals. The results supported the predictive performance of the models within the design space investigated. RSM optimization was designed to identify a formulation with favorable self-emulsification characteristics following HME; therefore, PS, ET, and PDI were selected as the optimization responses. Dissolution was evaluated separately as a performance measure of the RSM-selected formulation. Thus, the formulation optimized for the responses within the design space does not necessarily represent the formulation with the highest CBD dissolution. Another consideration for the formulation is its relatively high surfactant and co-surfactant content. F7 contains 32% *w*/*w* Gelucire^®^ 50/13 and 8% *w*/*w* PEG 300, representing a combined 40% *w*/*w* of the formulation. Gelucire^®^ 50/13 is a nonionic, water-dispersible surfactant used in lipid-based oral formulations and facilitates self-emulsification in aqueous media. However, selection of the RSM-optimized formulation in the present study was based primarily on physicochemical performance, including particle size, emulsification time, and PDI, rather than gastrointestinal tolerability.

Therefore, although the relatively high surfactant content contributed to favorable self-emulsification characteristics within the investigated design space, the potential gastrointestinal effects associated with the overall excipient exposure should be considered, particularly if the formulation is intended for repeated or chronic oral administration. The present study did not include gastrointestinal tolerability or repeated-dose in vivo evaluation; consequently, the long-term tolerability of this excipient combination at the proposed exposure remains to be established.

### 3.5. Characterization of Optimized S-SEDDS Formulation

#### 3.5.1. Emulsification Time

The time taken for the formulations to disperse in water and form an emulsion was recorded as the ET. The experimentally measured emulsification time of F7 was 37.3 ± 1.4 s, which was close to the model-predicted value of 36.12 s (Table 6). Under the standardized aqueous dispersion conditions employed in this study, F7 formed an emulsion after HME processing, indicating retention of self-emulsifying behavior after solidification. The comparatively short ET was consistent with the composition-response relationships established during optimization, which showed that lower sesame oil concentrations and a higher Gelucire^®^ 50/13-to-PEG 300 ratio promoted quicker emulsion formation. However, the present redispersion assessment was limited to the specified experimental conditions, and dilution robustness across different dilution ratios and media, as well as the time-dependent physical stability of the resulting emulsion, was not systematically investigated.

#### 3.5.2. Particle Size (PS), Zeta Potential (ZP) and Polydispersity Index Measurement (PDI)

Particle size and PDI are important characteristics of the emulsion formed following dilution in aqueous media for S-SEDDSs [50]. A low PS and PDI are desirable in the emulsion as it indicates that the nanoparticulate system has uniform dispersion and limited droplet size distribution [51]. After aqueous dilution, F7 produced droplets with a PS of 243.9 ± 7.14 nm and a PDI of 0.26 ± 0.05, similar to the expected values of 241.92 nm and 0.25, respectively (Table 6). The comparatively low PDI suggests a uniform droplet size distribution. These data, together with the short ET, demonstrate that F7 was able to easily redisperse following HME and make a rather homogeneous emulsion. The formulation also had a zeta potential of −24.0 ± 0.8 mV. The ZP value alone does not indicate stability of the emulsion; however, the absence of significant changes in PS, PDI, and ZP during the stability study demonstrates that the emulsion characteristics were not affected during storage. Solid-state characterization was then used to investigate the physical state of CBD and its compatibility with the formulation components. The PS and PDI results are interpreted within the context of the sample-preparation procedure, because the reconstituted S-SEDDS was filtered to remove insoluble Neusilin^®^ US2 before DLS analysis, larger lipid droplets or aggregates may also have been partially removed. Furthermore, the subsequent 100-fold dilution may influence interfacial and colloidal equilibrium. Therefore, the measured values characterize the filtered and diluted lipid dispersion under standardized analytical conditions and may not represent the absolute droplet-size distribution of the original undiluted S-SEDDS dispersion.

#### 3.5.3. Differential Scanning Calorimetry (DSC)

**Figure 2:**
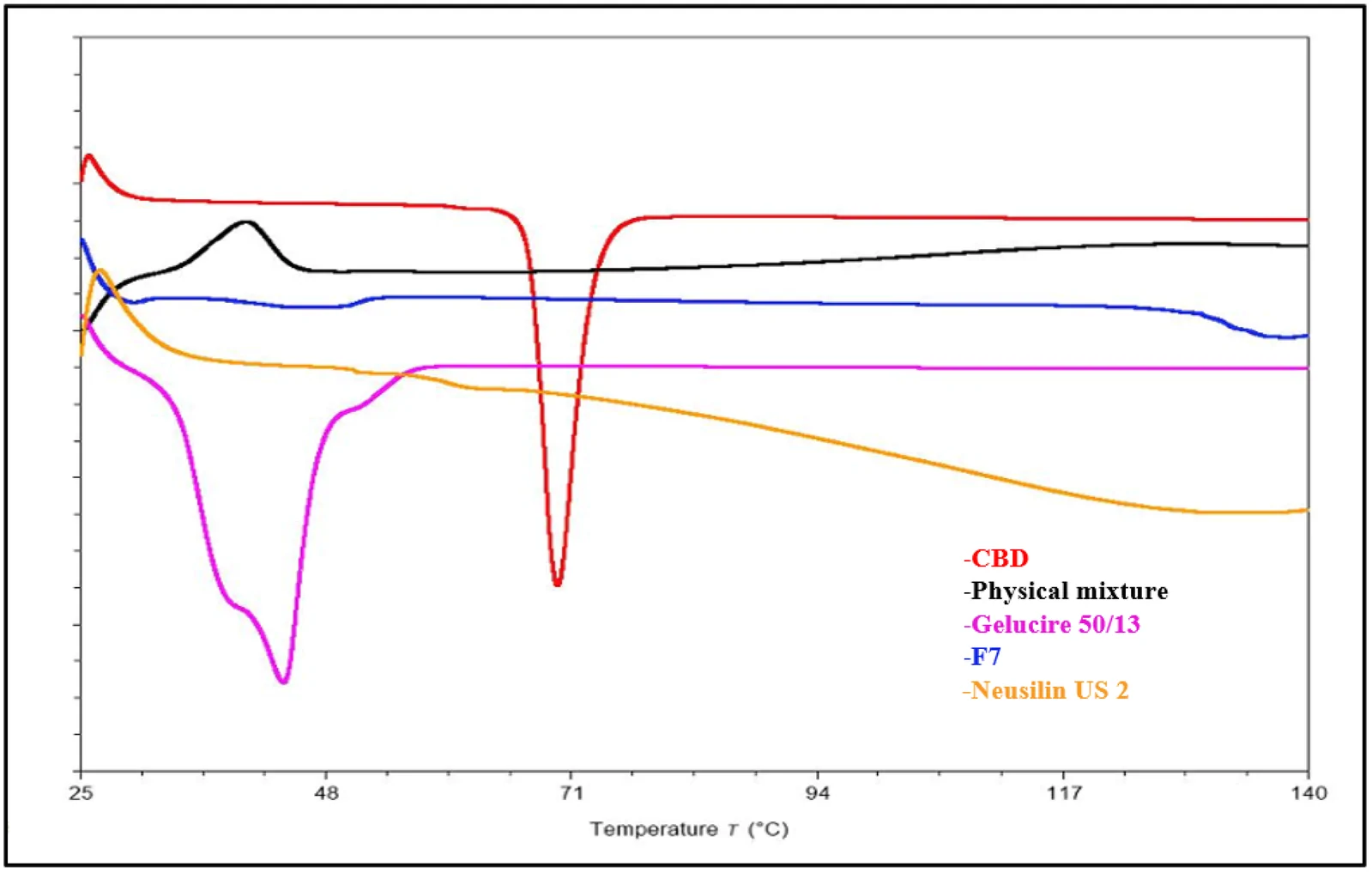
DSC thermogram of CBD, Gelucire^®^ 50/13, physical mixture, optimized formulation (F7) and Neusilin^®^ US2.

DSC is an essential tool for detecting both the endothermic (melting) and exothermic transitions like crystallization [52]. The DSC thermograms of pure CBD, excipients, the physical mixture, and the optimized S-SEDDS formulation (F7) are shown in Figure 2. Pure CBD displayed a sharp endothermic melting peak at approximately 67 °C, confirming its crystalline nature; this observation is comparable to the data found in the literature [53]. Gelucire^®^ 50/13 exhibited its own broad melting peak, while Neusilin^®^ US2 showed no defined endothermic peak, consistent with its amorphous nature. The distinct CBD melting peak was not directly visible in either the physical mixture or F7. In the physical mixture, this might be attributed to CBD dilution by the excipients. The lack of CBD crystallinity in F7 cannot be considered as evidence of amorphization of CBD or molecular dispersion. PXRD was consequently used to further assess the physical state of CBD in F7.

#### 3.5.4. Powder X-Ray Diffraction (PXRD)

**Figure 3:**
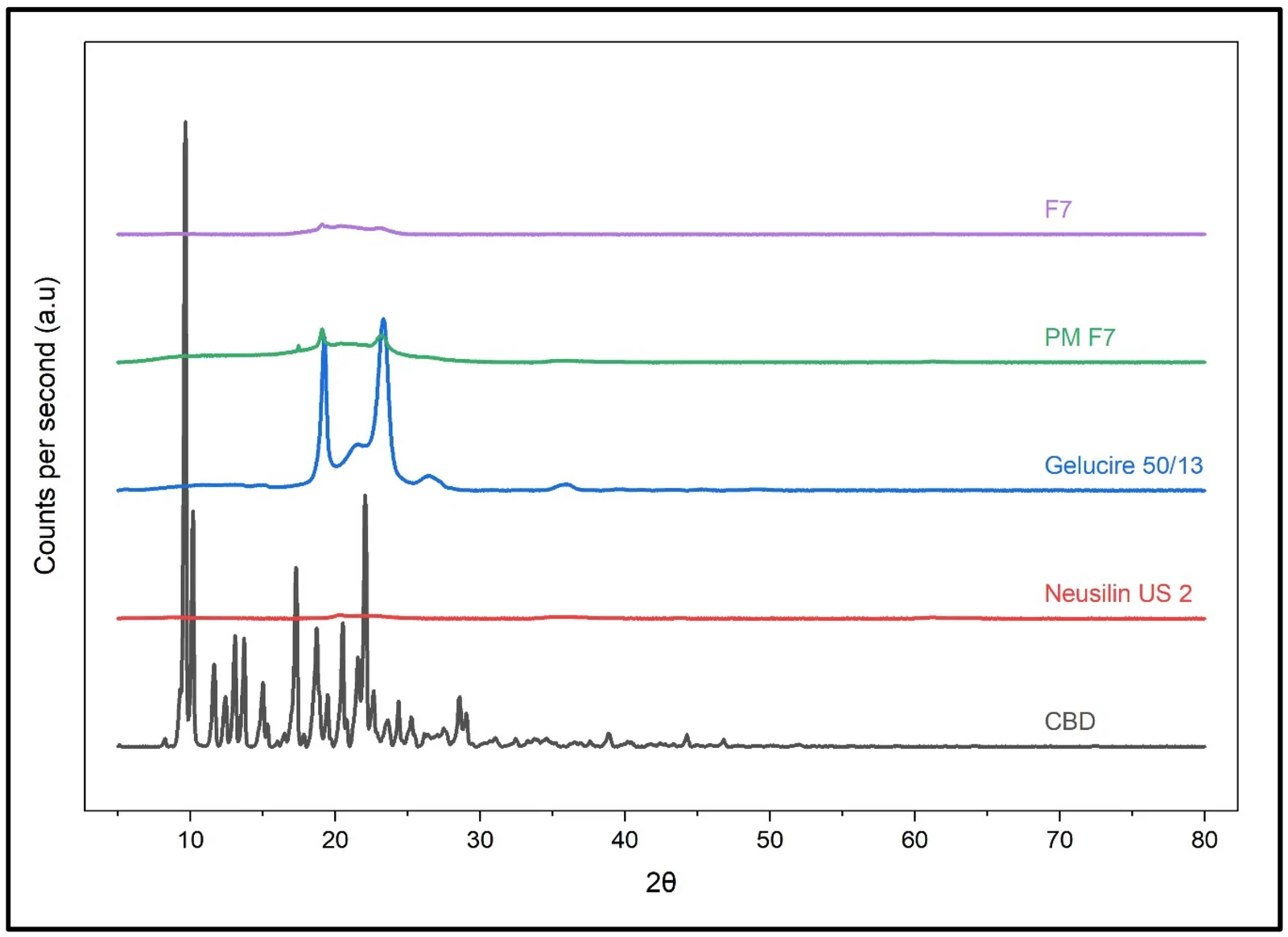
PXRD patterns of pure CBD, Neusilin^®^ US2, Gelucire^®^ 50/13, the physical mixture (PM F7), and RSM-optimized SEDDS formulation (F7).

PXRD was used to further examine CBD’s solid-state properties in formulation F7. Figure 3 shows that pure CBD has distinct diffraction peaks in the 2θ region (9–30°), indicating its crystalline structure. A similar diffraction pattern for pure CBD is found in previously published research [54]. In contrast, Neusilin^®^ US2 exhibited a broad diffraction pattern consistent with its largely amorphous structure, but Gelucire^®^ 50/13 showed discrete diffraction peaks at roughly 19° and 23°. The physical mixture (PM F7) demonstrated a significant reduction in the intensity of the distinctive CBD peaks, which might be due to drug dilution and overlap with the other formulation components. Following hot-melt extrusion, the characteristic CBD peaks were not observed in F7. This represents the conversion of CBD into an amorphous or molecularly dispersed state inside the solid SEDDS. These findings, combined with the DSC data, give additional evidence that HME changed the solid-state properties of CBD in the RSM-optimized formulation.

#### 3.5.5. Fourier Transform Infrared (FTIR) Analysis

**Figure 4:**
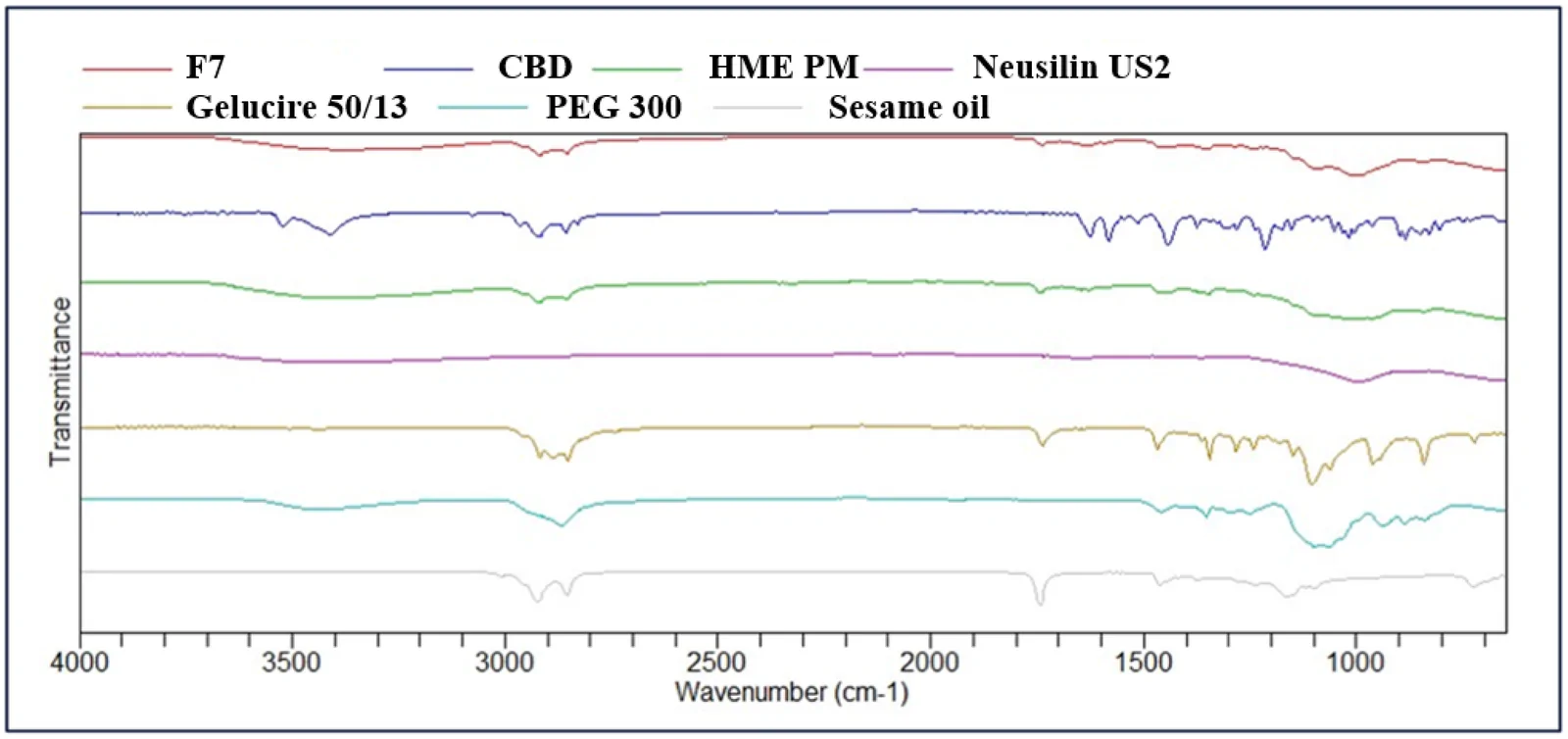
FTIR spectra of CBD, Gelucire^®^ 50/13, PEG 300, sesame oil, physical mixture, F7 and Neusilin^®^ US2.

FTIR spectroscopy was used to assess potential interactions between CBD and the excipients in the optimized S-SEDDS formulation (F7) (Figure 4). The spectra of pure CBD exhibited its characteristic bands, including the O–H stretching around 3350 cm^−1^, C–H stretching near 2920–2850 cm^−1^, and aromatic and C=C stretching vibrations between 1600 and 1500 cm^−1^. The results obtained were consistent and in alignment with previously published research [55,56]. These distinct CBD bands were visible in the physical mixture but appeared reduced in intensity due to dilution with other excipients. In the optimized formulation (F7), no major shifts in the characteristic functional group regions or formation of new bands were observed in the FTIR spectrum. These results indicate that no obvious changes in the functional groups were detected by FTIR under the test conditions. However, FTIR alone cannot be used to rule out potential chemical degradation.

#### 3.5.6. Drug Content

The optimized S-SEDDS formulation exhibited good content uniformity, with CBD content of 98.2 ± 1.9% (n = 3) following HME. The low variation in drug content among samples collected from different sections of the batch confirms consistent drug distribution and indicates that the HME process uniformly distributed the CBD in the formulation. However, because the HPLC method used for CBD quantification was not established as a stability-indicating method capable of detecting CBD from all potential degradation products, the assay results alone cannot exclude the formation of low levels of degradation products during HME.

### 3.6. In Vitro Dissolution Testing

**Figure 5:**
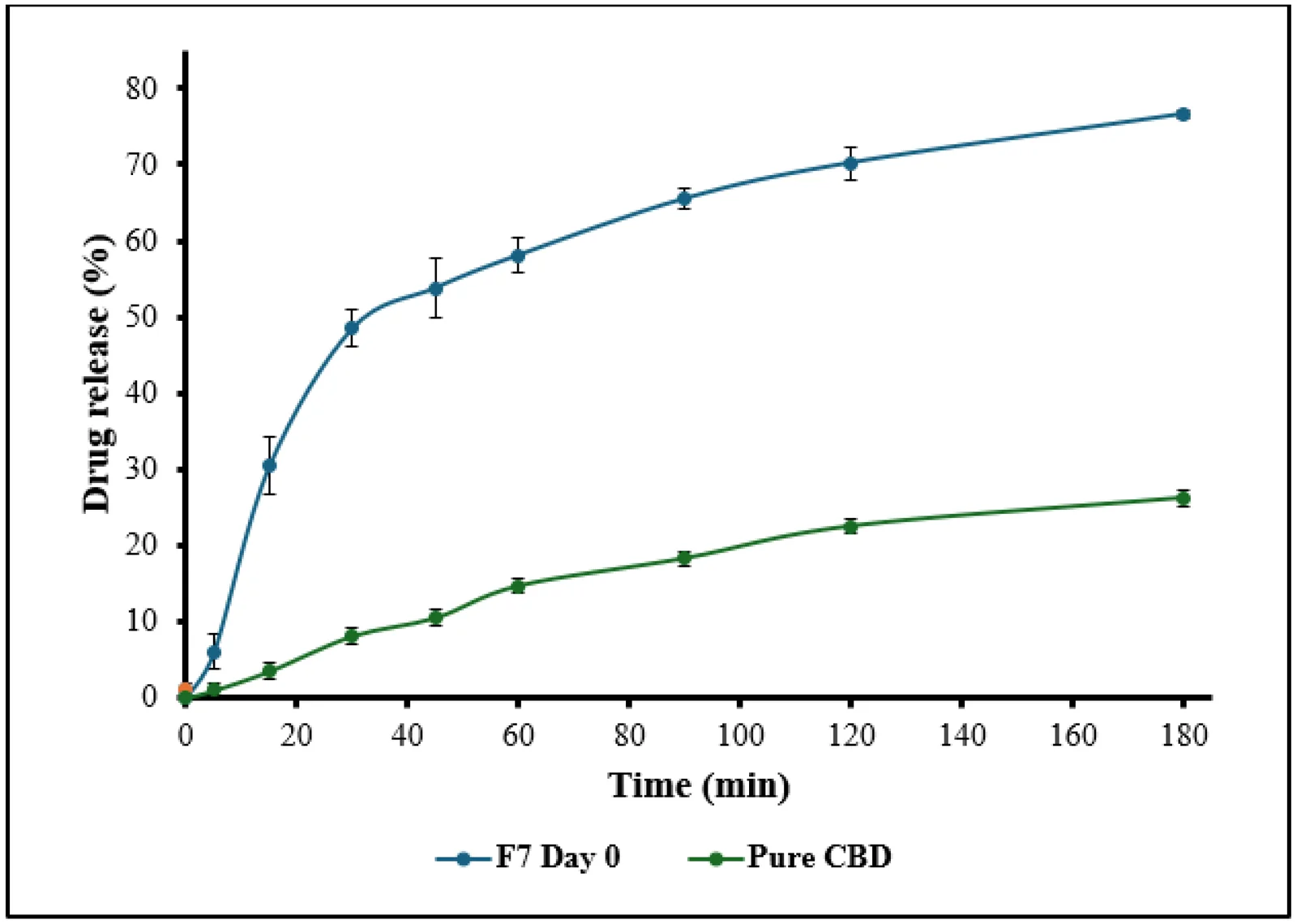
Dissolution profile of formulation F7 and pure CBD at Day 0.

In vitro drug release for the HME-processed S-SEDDS formulation (F7) was evaluated using the dissolution method described previously in the Materials and Methods Section. The resulting percent release profile vs. time plot is presented in Figure 5. In comparison to pure CBD, the S-SEDDS formulation (F7) showed a faster and improved dissolution profile. F7 demonstrated effective self-emulsification and drug release, releasing around 35% of the CBD in the first 15 min, reaching ~76% release at 180 min. Pure CBD, on the other hand, had a slower dissolution profile, releasing just about 10% after 45 min and 28% after 180 min. Despite the substantial improvement in CBD release compared with the pure drug, complete drug release was not achieved from F7. One possible explanation is the use of Neusilin^®^ US2 as the solid carrier. Neusilin^®^ US2 is a porous synthetic magnesium aluminosilicate with a high surface area and adsorption capacity [57]. Therefore, retention of a fraction of CBD and/or lipid formulation components within the porous carrier matrix could potentially contribute to incomplete drug release. However, this mechanism was not directly investigated in the present study. Since no alternative solid carriers were evaluated and the distribution or retention of CBD within Neusilin^®^ US2 was not directly characterized, the incomplete release cannot be conclusively attributed to carrier adsorption. Further studies comparing different solid carriers and directly investigating drug distribution and retention within the carrier matrix would be required to establish the mechanism responsible for the unreleased fraction. Similar results of incomplete or partial release from S-SEDDSs containing Neusilin^®^ US2 have also been reported in previous studies [58,59,60]. The increased CBD release from F7 compared with pure CBD demonstrates improved release under the dissolution conditions employed in this study. However, these conditions were intended primarily for comparative formulation assessment and should not be considered fully biorelevant. In particular, the phosphate buffer containing 0.1% Tween 80 does not reproduce the complex composition and dynamic conditions encountered in the gastrointestinal tract, and the presence of Tween 80 may increase CBD solubilization. Furthermore, because F7 is a lipid-based formulation, gastrointestinal digestion of the lipid components may alter the colloidal environment and consequently influence CBD solubilization, precipitation, and availability for absorption. In vitro lipolysis and dissolution studies using biorelevant simulated gastrointestinal media were not performed in the present investigation. Therefore, the observed improvement in CBD release cannot be directly extrapolated to in vivo performance. Future studies incorporating simulated gastric and intestinal conditions and lipid-digestion models are warranted to establish the fate of CBD during gastrointestinal processing and provide a more physiologically relevant assessment of formulation performance. Although F7 exhibited favorable self-emulsification characteristics and substantially greater CBD release than the pure drug, the present study was not designed to establish a quantitative relationship between particle size, PDI, emulsification time, and dissolution performance. Dissolution testing was performed for the optimized formulation and pure CBD rather than across all CCD formulations; therefore, a correlation analysis between the self-emulsification responses and CBD release could not be reliably performed. Systematic dissolution testing across formulations in the experimental design space would be required to determine whether these parameters quantitatively correlate with CBD release.

### 3.7. Short-Term Stability Testing

**Figure 6:**
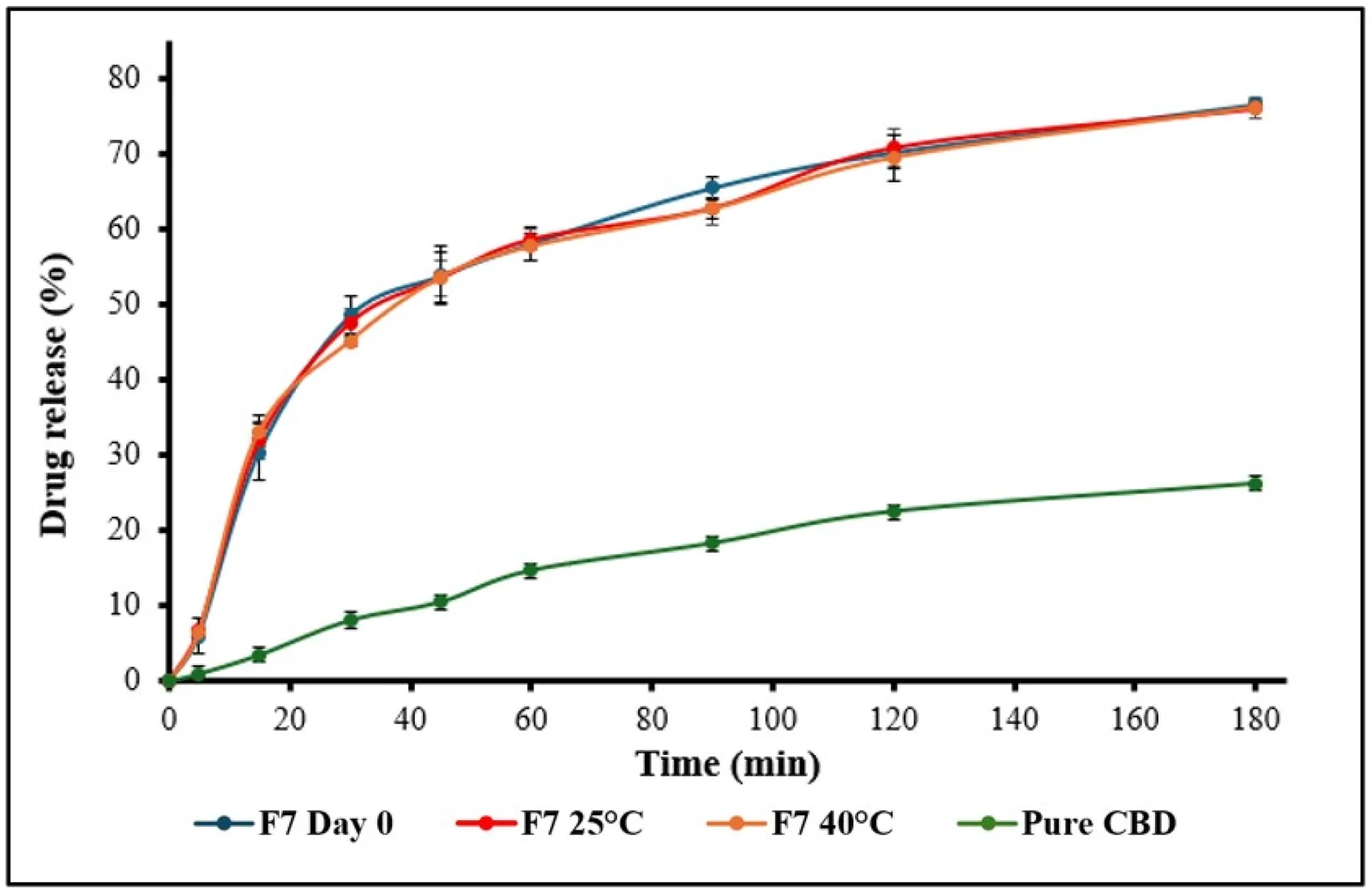
Dissolution profiles of F7 following 90 days of storage at 25 °C and 40 °C.

The optimized formulation F7 was assessed for appearance, PS, PDI, ZP, drug content, and dissolution behavior over 90 days under (25 ± 2 °C) and (40 ± 2 °C) storage conditions. The detailed results for particle size, PDI, zeta potential, drug content, and dissolution are presented in Table 10 and Figure 6. No visible changes in color or physical appearance were observed throughout the study. PS, PDI, and ZP remained stable and no apparent changes were observed over time. Likewise, drug content and dissolution profiles exhibited minimal variation, indicating that F7 maintained its physicochemical characteristics and release performance during storage. In addition, hot-melt extrusion of the SEDDSs with the solid carrier Neusilin^®^ US2 produced free-flowing extrudates; the carrier helped maintain the physical stability of the formulation during storage. The porous nature of the carrier could have also helped by immobilizing the lipid components and reducing the possibility of phase separation or recrystallization of the drug during the study at both storage conditions. However, this mechanism was not directly investigated in the present study. The study was conducted under temperature-controlled conditions without controlling relative humidity. Therefore, these results are considered preliminary short-term stability data rather than evidence of long-term stability under ICH-defined storage conditions. Future studies under controlled temperature and relative humidity for longer storage periods are needed to establish the long-term stability of the formulation.

## 4. Conclusions

In the current study, CBD-loaded S-SEDDSs were successfully formulated by HME and optimized using Response Surface Methodology (RSM) and Central Composite Design (CCD). The RSM-optimized formulation, produced via hot-melt extrusion, exhibited a favorable particle size, quick emulsification, and a narrow size distribution. The characterization studies, including DSC, FTIR, drug content analysis, and in vitro dissolution, confirmed efficient drug mixing, substantial reduction in CBD crystallinity, and significantly improved dissolution profile compared to pure CBD. Stability studies further demonstrated that the formulation maintained its physicochemical attributes, drug content, and release behavior for three months. Overall, these results indicated the significance of RSM in understanding and improving the link between formulation composition and performance of HME-processed CBD S-SEDDSs. Future studies comparing alternative solid carriers, biorelevant dissolution performance and long-term stability testing under controlled temperature and relative humidity are needed to further evaluate formulation performance.

## Figures and Tables

**Table 1:** Independent and dependent variables with their coded levels used in the Central Composite Design (CCD).

Independent Variables	Coded Levels				
	−α	−1	0	+1	+α
**X_1_; Oil Concentration (% *w*/*w*)**	7.92	10	15	20	22.07
**X_2_; Surfactant to Co-Surfactant Ratio**	1.58:1	2:1	3:1	4:1	4.41:1
**Dependent Variables**	Y_1_; particle size after aqueous dispersion (nm)				
	Y_2_; emulsification time (s)				
	Y_3_; polydispersity index (PDI)				

(α) represents axial distance from the center point; coded levels −1, 0, and +1 represent the low, center, and high levels, respectively, while −α and +α represent the axial levels.

**Table 2:** Composition of CBD S-SEDDS formulations generated according to the Central Composite Design (% *w*/*w* of total formulation).

Formulation	Run	Drug Load	Neusilin^®^ US2	Sesame Oil	Gelucire^®^ 50/13	PEG 300
**F1**	2	10	40	20	20	10
**F2**	10	10	40	15	26.25	8.75
**F3**	9	10	40	15	26.25	8.75
**F4**	11	10	40	15	26.25	8.75
**F5**	8	10	40	15	28.54	6.46
**F6**	4	10	40	20	24.0	6.0
**F7**	3	10	40	10	32.0	8.0
**F8**	5	10	40	7.92	31.55	10.52
**F9**	1	10	40	10	26.67	13.33
**F10**	6	10	40	22.07	20.95	6.98
**F11**	7	10	40	15	21.46	13.54

**Table 3:** Excipient screening study of CBD in different oils, surfactants, and co-surfactants.

Excipient	Screening Outcome
**Tween 20**	(+)
**Sesame Oil**	(+)
**Propylene Glycol**	(−)
**Castor Oil**	(+)
**Cremophor^®^ EL**	(+)
**Poloxamer 188**	(−)
**Kolliphor^®^ RH 40**	(−)
**Comparitol ATO 888**	(−)
**Gelucire^®^ 44/14**	(−)
**Gelucire^®^ 50/13**	(+)
**Gelucire^®^ 48/16**	(−)
**Gelucire^®^ 59/14**	(−)
**PEG 300**	(+)
**Kolliphor^®^ HS 15**	(−)

(+) No visible drug precipitation/phase separation observed on cooling; (−): visible drug precipitation/phase separation observed on cooling.

**Table 4:** Design runs of hot-melt-extruded CBD S-SEDDSs and their responses in terms of particle size and time and PDI (mean, n = 3).

Formulation	Run	PS (nm)	ET (sec)	PDI
**F1**	2	345.6	75.1	0.4
**F2**	10	250.1	52.6	0.3
**F3**	9	251.5	52.9	0.3
**F4**	11	249.8	52.4	0.3
**F5**	8	245.4	45.2	0.3
**F6**	4	264.1	50.3	0.3
**F7**	3	242.5	35.8	0.3
**F8**	5	240.9	37.2	0.2
**F9**	1	266.7	57.5	0.4
**F10**	6	310.4	55.2	0.4
**F11**	7	331.6	80.5	0.4

**Table 5:** ANOVA and model summary statistics for the quadratic model of particle size.

Source	Globule Size (Y_1_, nm)			
	Sum of Squares	Degree of Freedom	F-Value	*p*-Value
**Model**	14,417.41	5	314.74	<0.0001
**A—Oil Concentration**	4939.58	1	539.17	<0.0001
**B—Surfactant: Co-Surfactant Ratio**	6472.30	1	706.48	<0.0001
**AB**	820.82	1	89.60	0.0002
**A^2^**	814.08	1	88.86	0.0002
**B^2^**	1916.37	1	209.18	<0.0001
**Residuals**	45.81	5		
**Lack of Fit**	44.16	3	17.88	0.0534
**Pure Error**	1.65	2		
**Total**	14,463.22	10		
**R^2^**	0.9968			
**Adjusted R^2^**	0.9937			
**Predicted R^2^**	0.9780			
**C.V. %**	1.11			
**Adequate Precision**	48.837			

**Table 6:** ANOVA and model summary statistics for the quadratic model of emulsification time.

Source	Emulsification Time (Y2, sec)			
	Sum of Squares	Degree of Freedom	F-Value	*p*-Value
**Model**	1866.40	5	268.11	<0.0001
**A—Oil Concentration**	410.26	1	249.67	<0.0001
**B—Surfactant: Co-Surfactant Ratio**	1166.79	1	838.06	<0.0001
**AB**	2.12	1	1.52	0.2723
**A^2^**	54.33	1	39.02	0.0015
**B^2^**	154.22	1	110.77	0.0001
**Residuals**	6.96	5		
**Lack of Fit**	6.72	3	18.50	0.0517
**Pure Error**	0.2421	2		
**Total**	1873.36	10		
**R^2^**	0.9963			
**Adjusted R^2^**	0.9926			
**Predicted R^2^**	0.9472			
**C.V. %**	2.18			
**Adequate Precision**	50.3960			

**Table 7:** ANOVA and model summary statistics for the linear model of polydispersity index (PDI).

Source	PDI (Y3)			
	Sum of Squares	Degree of Freedom	F-Value	*p*-Value
**Model**	0.0188	2	16.82	0.0014
**A—Oil Concentration**	0.0125	1	22.30	0.0015
**B—Surfactant: Co-Surfactant Ratio**	0.0063	1	11.34	0.0098
**Residuals**	0.0045	8		
**Lack of Fit**	0.0039	6	2.39	0.3244
**Pure Error**	0.0005	2		
**Total**	0.0233	10		
**R^2^**	0.8079			
**Adjusted R^2^**	0.7598			
**Predicted R^2^**	0.5835			
**C.V. %**	7.24			
**Adequate Precision**	10.9529			

**Table 8:** Criteria of variables and responses for the optimization step.

Variable	Goal	Lower Limit	Upper Limit
**Oil concentration (% *w*/*w*)**	In range	10	20
**Surfactant–co-surfactant ratio**	In range	2.0	4.0
**Particle size (nm)**	Minimize	200	250
**Emulsification time (s)**	Minimize	30	60
**PDI**	Minimize	0.20	0.30

**Table 9:** Results of validation trials of the proposed formulation (mean, n = 3).

Response	Predicted Value	Observed Value	Prediction Error (%)	95% CI (Low)	95% CI (High)
**Particle size (nm)**	241.92	243.9	0.82	235.77	248.79
**Emulsification time (s)**	36.12	37.3	3.27	33.72	38.53
**PDI**	0.25	0.26	4.00	0.22	0.29

**Table 10:** Stability data for optimized formulation F7 at 25 °C and 40 °C (mean ± SD, n = 3).

Formulation	Storage (°C)	Time (Day)	Particle Size (nm)	Polydispersity Index	Zeta Potential (mV)	Drug Content (%)	Dissolution (%)
**F7 (Optimized Formulation)**	25	0	243.9 ± 7.14	0.26 ± 0.05	−24.0 ± 0.8	98.15 ± 1.94	76.6 ± 0.6
		30	243.7 ± 5.42	0.26 ± 0.03	−23.9 ± 0.7	97.8 ± 0.58	76.16 ± 0.37
		60	243.5 ± 5.67	0.27 ± 0.04	−24.2 ± 0.4	97.47 ± 0.48	76.02 ± 0.6
		90	243.8 ± 5.91	0.27 ± 0.04	−24.1 ± 0.7	97.40 ± 0.62	76.0 ± 1.3
	40	30	244.0 ± 5.13	0.26 ± 0.02	−24.0 ± 0.8	97.85 ± 0.78	76.12 ± 0.06
		60	244.1 ± 5.49	0.27 ± 0.02	−24.3 ± 0.5	97.78 ± 0.84	75.92 ± 0.69
		90	244.2 ± 5.88	0.27 ± 0.04	−24.4 ± 0.6	97.37 ± 0.58	76 ± 0.2

## Data Availability

The authors declare that all data will be available upon request.

## Abbreviations

The following abbreviations have been used in the manuscript:

CBD	Cannabidiol
THC	Tetrahydrocannabinol
LBDDS	Lipid-based drug delivery systems
SEDDSs	Self-emulsifying drug delivery systems
o/w	Oil in water
GI	Gastrointestinal
HME	Hot-melt extrusion
PAT	Process Analytical Technology
S-SEDDS	Solid-SEDDS
OFAT	One factor at a time
RSM	Response Surface Methodology
CCD	Central Composite Design
ET	Emulsification time
PS	Particle size
ZP	Zeta potential
PDI	Polydispersity index
DSC	Differential scanning calorimetry
PXRD	Powder X-Ray Diffraction
FTIR	Fourier Transform Infrared
ATR	Attenuated Total Reflectance
HPLC	High-performance liquid chromatography
